# Identification of Cancerlectins Using Support Vector Machines With Fusion of G-Gap Dipeptide

**DOI:** 10.3389/fgene.2020.00275

**Published:** 2020-04-03

**Authors:** Lili Qian, Yaping Wen, Guosheng Han

**Affiliations:** Key Laboratory of Intelligent Computing and Information Processing of Ministry of Education and Hunan Key Laboratory for Computation and Simulation in Science and Engineering, Xiangtan University, Xiangtan, China

**Keywords:** cancerlectins, g-gap dipeptide, feature selection, analysis of variance, support vector machine

## Abstract

The cancerlectin plays an important role in the initiation, survival, growth, metastasis, and spread of cancer. Therefore, to study the function of cancerlectin is greatly significant because it can help to identify tumor markers and tumor prevention, treatment, and prognosis. However, plenty of studies have generated a large amount of protein data. Traditional prediction methods have been unable to meet the needs of analysis. Developing powerful computational models based on these data to discriminate cancerlectins and non-cancerlectins on a large scale has been treated as one of the most important topics. In this study, we developed a feature extraction method to identify cancerlectins based on fusion of g-gap dipeptides. The analysis of variance was used to select the optimal feature set and a support vector machine was used to classify the data. The rigorous nested 10-fold cross-validation results, demonstrated that our method obtained the prediction accuracy of 83.91% and sensitivity of 83.15%. At the same time, in order to evaluate the performance of the classification model constructed in this work, we constructed a new data set. The prediction accuracy of the new data set reaches 83.3%. Experimental results show that the performance of our method is better than the state-of-the-art methods.

## Introduction

Cell recognition is the central event of various biological phenomena. The combination of cell surface molecular selectivity with other molecules is an important link in cell development and differentiation, such as fertilization, embryogenesis, immune defense, pathogen infection, and pathogenicity. Abnormal cell recognition may lead to diseases, such as defects in leukocyte and platelet adhesion, which can lead to the recurrence of bacterial infections and mucosal bleeding, respectively. In addition, abnormal cell recognition is considered to be the basis of uncontrolled cell growth and movement, which is the characteristic of tumor transformation and metastasis (Sharon and Lis, 1989).

Lectin is one of the cell recognition molecules. It is a biological molecule that specifically recognizes and binds the carbohydrate components existing in other proteins (Kumar and Panwar, 2011). Most lectins have high specificity and selectivity in identifying sugar molecules present in other proteins (Lis and Sharon, 1998). According to their affinity with monosaccharides, these glycoproteins can be divided into five categories: mannose, N-acetylglucosamine, galactose/N-acetylgalactosamine, fucose, and sialic acid, which represent a group of heterogeneous oligomeric proteins (Kumar and Panwar, 2011). It has been found that lectins are involved to a variety of biological processes, such as maintaining the dynamic balance of cell proliferation and apoptosis, cell differentiation, cell adhesion and migration, cell-extracellular matrix interaction, host-pathogen interaction, cell-cell recognition, complement activation pathway, immune defense, and regulation of inflammatory response (Lin et al., 2015). Lectin molecules provide biological scripts to decipher complex codes in sugar groups (Damodaran et al., 2008). Therefore, lectins are often used as diagnostic and therapeutic tools in many fields such as cell biology, biochemistry, and immunology.

Cancerlectins are a group of lectins which are closely related to cancer (Kumar and Panwar, 2011). Lectin participates in serum-glycoprotein transformation and innate immune response, and has a special correlation with the growth and metastasis of tumors (Damodaran et al., 2008). Some evidences suggest that tumor cell agglutinin is involved in cell interactions, such as adhesion, cell growth, differentiation, metastasis and infection of cancer cells (Lis and Sharon, 1998). Whether basic research or clinical application, cancerlectins has been widely used in cancer research (Lai et al., 2017). For example, sialic acid-bound immunoglobulin lectin-9 is a neutrophil-specific expression that binds to sugar molecules on the surface of cancer cells, regulates immune response and promotes or inhibits tumor progression; spiral hemagglutinin is an effective prognostic indicator of colorectal cancer, etc. (Kumar and Panwar, 2011). The effect of lectins on the immune system by altering the production of various interleukins has been well-documented. There is also data showing that some lectins down-regulate the activity of telomere, thereby inhibiting angiogenesis (Choi et al., 2004; De Mejía and Prisecaru, 2005). Cancerlectins can induce cytotoxicity, apoptosis, and inhibit tumor growth by binding to receptors on the surface of cancer cells. It can be used as a therapeutic method for cancer treatment. Cancer is the second leading cause of death in the world. Therefore, the screening of specific lectins from a large number of lectins is of great significance not only for the discovery of tumor markers and cancer treatment, but also for better understanding and conquering cancer (Balachandran et al., 2017).

A plenty of studies have generated a large amount of protein data, using traditional biological experiments to predict and analyze the function of proteins is not only time-consuming but also laborious. Based on these data, it is one of the most important topics to predict a cancerous substance by establishing a powerful computational model to identify cancerous and non-cancerous substances on a large scale. The description of the characteristics of the protein sequence method contains a lot of information, such as the chemical and physical properties of amino acids, sequence characteristics, feature extraction algorithm for classification algorithm which has great impact on the design and the classification of results. Too few protein sequence characteristics will result in the loss of important information of protein sequence and affect the classification results, and therefore dimension disaster, conversely, there is no guarantee of the classification efficiency of the model. Therefore, how to conduct efficient feature fusion and establish appropriate mathematical expression methods and similarity measurement standards is an important problem.

### Feature Extraction Based on Sequence Information

Nakashima et al. (1986) proposed amino acid composition to study protein folding. One of the most basic algorithms for extracting features of protein sequence is amino acid composition, which represents the occurrence frequency of each of the 20 common amino acids in the protein sequence and converts the protein sequence into a 20-dimensional feature vector. Yu et al. (2004) proposed using k peptide component information to represent protein sequences. Feng et al. (2013) proposed a Naïve Bayes-based method to predict antioxidant proteins using amino acid compositions and dipeptide compositions.

### Feature Extraction Based on Physical and Chemical Properties of Amino Acids

Bu et al. (1999) proposed an autocorrelation function algorithm, which is a description method based on Amino Acid Residue Index (Kawashima et al., 1999), for the study of protein structure predetermination. Chou (2001) proposed the pseudo-amino acid composition method, including sequence order information other than amino acid composition.

### Feature Extraction Based on Protein Evolution Information

Evolutionary information is one of the most important information of protein functional annotation in biological analysis, reflecting the sequence conservation of amino acids at each site of protein sequence in the evolutionary process (Xu et al., 2015). Evolutionary information of proteins mainly relies on positional specificity score matrix (PSSM) (An et al., 2016).

In the published research work, Kumar and Panwar (Kumar and Panwar, 2011) integrated PROSITE domain information with PSSM, developed a support vector machine model, and obtained MCC value of 0.38 with an accuracy of 69.09%; Lin et al. (2015) developed a sequence-based method to distinguish cancerlectins from non-cancerlectins, and used ANOVA to select the optimal feature subset. The accuracy of the method is 75.19%; Zhang et al. (2016) proposed a classification model based on random forest, the accuracy of the method is 70%; Lai et al. (2017) proposed a new method of feature expression based on amino acid sequence, and binomized it. In the jackknife cross-validation, the accuracy is 77.48%. Han et al. (2014) proposed a two-stage multi-class support vector machine combined with a two-step optimal feature selection process for predicting membrane protein types. Anh et al. (2014) propose a kernel method, named as SSEAKSVM, predicting protein structural classes for low-homology data sets based on predicted secondary structures. Balachandran et al. (2018) proposed a support vector machine (SVM)-based PVP predictor, called PVP-SVM, which was trained with 136 optimal features. Runtao et al. (2018) proposed a computational method based on the RF (Random Forest) algorithm for identifying cancerlectins, and achieves a sensitivity of 0.779, a specificity of 0.717, an accuracy of 0.748. These methods have obtained quite good results, but the accuracy still needs to be improved. In this work, we constructed a new classification system of protein sequences, and the relatively better result was obtained on the benchmark dataset and the independent test dataset.

## Methods

### Dataset

Data acquisition is the first step of data analysis. The benchmark dataset is not only the database of algorithm learning, but also the cornerstone of classification model. Constructing a good benchmark data set also plays an important role in the performance of classification model (Lin and Chen, 2010). In order to compare objectively with the existing research results, the dataset used in this work was widely used which was constructed by Kumar and Panwar (Kumar and Panwar, 2011).

The benchmark dataset contains both positive and negative samples. The original data were downloaded from the CancerLectinDB database (Damodaran et al., 2008), removing duplicated sequences and sequences without experimental evidence, or containing non-standard amino acids, and 385 proteins were obtained to form a positive subset (Lin et al., 2015). Using the keyword “lectins” search in UniProt database, deleting the sequences labeled “similarity,” “fragment,” “hypothesis,” and “possibility,” a negative subset containing 820 proteins was constructed (Kumar and Panwar, 2011; Lin et al., 2015). If the designed data sets contain highly similar sequences, misleading results with high prediction accuracy will be obtained, thus reducing the generalization ability of the model. In order to remove homologous sequences from the benchmark dataset, the CD-HIT program was employed with 50% as the sequence identity cutoff to exclude any protein/peptide sequences with more than 50% paired sequence in the benchmark dataset (Lin et al., 2015). The benchmark dataset *S* can be formulated as follows:

$$\begin{array}{l} S=S_{+}\cup S_{-} \end{array}$$

where the positive subset *S*_+_ contains 178 cancerlectin samples, the negative subset *S*_contains 226 non-cancerlectin samples, thus, the benchmark dataset *S* contains 404 samples. The benchmark dataset is available at https://github.com/hangslab/cancerlectins.

### Feature Extraction Method

When using the machine learning method, protein sequences need to be transformed into numerical vectors representing the characteristics of protein sequence. The extracted features need not only to retain the sequence information of proteins to the greatest extent, but also to have a greater correlation with protein classification.

**Figure 1:**
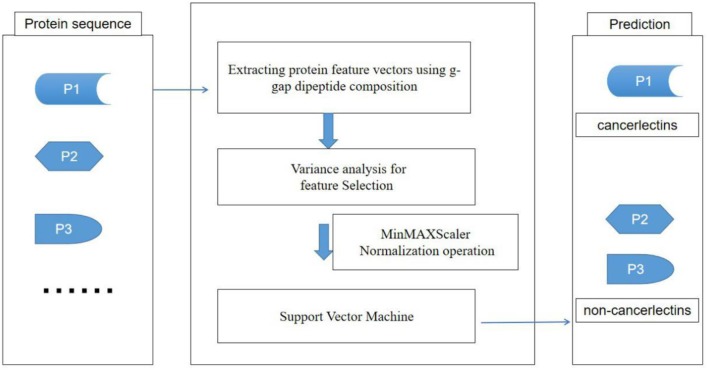
The flowchart of our method.

The sequence of amino acids in protein sequence is the basis of protein biological function. The dipeptide composition is the condition of *k* = 2 in the feature extraction method of k-peptide composition (Yu et al., 2004; Lin and Chen, 2010). The dipeptide composition can only reflect the correlation of adjacent amino acids in protein sequence. Generally speaking, the intrinsic properties of protein sequences may be precipitated in higher-level residue relationships. In the tertiary structure of proteins, the two amino acids separated from the original sequence may be very close in space, which means that the g-gap dipeptide composition (Sharma and Paliwal, 2008; Lin et al., 2015) contains more information about protein sequences than the dipeptide composition. In this paper, we developed a feature extraction method of fusion g-gap dipeptide component, Figure 1 is the flow chart of the model construction.

The *g*-gap dipeptide composition transforms each protein sequence into a feature vector. For each *g* value, a 400-dimensional feature vector (20^*^20) will be generated. The range of g is [0,9]. g = *g*_*h*_,g_*h*_ = *h*,*h* ∈ [0, 9] is used to distinguish the frequency of *g*-gap dipeptides with different values of g. We transformed a cancerlectin or non-cancerlectin protein sample *P* with *L* amino acids into an input vector of 4,000 dimensions, defined as follows:

$$\begin{array}{l} F_{4000}={\left[f_{1}^{0},\cdots \;,f_{400}^{0},f_{1}^{1},\cdots f_{400}^{1},\cdots \;,f_{u}^{g_{h}},{\cdots \;,f}_{1}^{9},\cdots \;,\;f_{400}^{9}\;\right]}^{T} \end{array}$$

where the $f_{u}^{g_{h}}$ is the frequency of the *u*-th (*u* = 1, 2, ⋯, 400) *g*_*h*_-gap dipeptide and calculated by

$$\begin{array}{l} f_{u}^{g_{h}}=\frac{n_{u}^{g_{h}}}{\sum_{u=1}^{400}n_{u}^{g_{h}}} \end{array}$$

where $n_{u}^{g_{h}}$denote the number of the *u*-th *g*_*h*_-gap dipeptide in a protein. Note that when *g* = 0, the *g*-gap dipeptide will degenerate to the adjoining dipeptide composition.

The class labels corresponding to each feature vector are represented by *t*, *t* ∈ {0, 1},1 represents positive sample and 0 represents negative samples. Finally, a 404^*^4,000 feature matrix was obtained.

### Feature Selection

When the number of features is large, there may be unrelated features, or interdependence between features, which easily leads to the time-consuming process of analyzing features and training models. The more the number of features, the more likely it is to cause “dimension disaster,” the more complex the model will be, and its generalization ability will decline. Feature selection can eliminate irrelevant or redundant features, reduce the number of features, improve the accuracy of the model and reduce the running time. On the other hand, the model is simplified by selecting truly relevant features, which makes it easy for researchers to understand the process of data generation.

Influenced by the collinearity of sample features, the results of linear discriminant analysis are poor (Lin et al., 2013), and the use of binomial distribution will lead to a high-dimensional feature vector (Yanyuan et al., 2018), which consumes a lot of computing time and may lead to over-fitting. After comparison, the feature selection method used in this paper is variance analysis (Lin et al., 2015). The variance analysis decomposes the difference of samples at the level of known influencing factors into intra-group variance and inter-group variance. The intra-group variance is not affected by the level of influencing factors, but mainly sampling error. The variance between groups is influenced by the level of factors, which is the essential difference between samples. The characteristic variance is measured by calculating the ratio F of variance between feature groups and variance within the group. The *F*-value of the *u*-th feature in the benchmark dataset is defined as follows:

$$\begin{array}{l} F\left(u\right)=\frac{S_{A}^{2}\left(u\right)}{S_{E}^{2}\left(u\right)} \end{array}$$

where $S_{A}^{2}\left(u\right)$ is the sample variance between groups, $S_{E}^{2}\left(u\right)$ is the sample variance within groups. They are given by:

$$\begin{array}{l} \{\begin{array}{l} S_{A}^{2}(u)=\frac{SS_{A}(u)}{df_{A}}\\ S_{E}^{2}(u)=\frac{SS_{E}(u)}{df_{E}} \end{array} \end{array}$$

where *SS*_*A*_(*u*) is sum of squares between groups and *SS*_E_(*u*) is sum of squares within groups, which can be calculated by:

$$\begin{array}{l} \{\begin{array}{c} SS_{A}(u)=\sum_{i=1}^{K}m_{i}{\left(\frac{\sum_{j=1}^{m_{i}}f_{u}^{g_{h}}\left(i,j\right)}{m_{i}}-\frac{\sum_{i=1}^{K}\sum_{j=1}^{m_{i}}f_{u}^{g_{h}}\left(i,j\right)}{\sum_{i=1}^{K}m_{i}}\right)}^{2}\\ SS_{E}(u)=\sum_{i=1}^{K}\sum_{j=1}^{m_{i}}{\left(f_{u}^{g_{h}}\left(i,j\right)-\frac{\sum_{j=1}^{m_{i}}f_{u}^{g_{h}}\left(i,j\right)}{m_{i}}\right)}^{2} \end{array} \end{array}$$

where $f_{u}^{g_{h}}\left(i,j\right)$ is the frequency of the *u*-th *g*_*h*_-gap dipeptide of the *j*-th sample in the *i*-th group; *m*_*i*_denotes the number of samples in the *i*-th group (here *m*_1_ = 178, *m*_2_ = 226).

*df*_*A*_ and *df*_*E*_ are degrees of freedom for the sample variance between groups and the sample variance within groups, respectively. They can be calculated by:

$$\begin{array}{l} \{\begin{array}{c} df_{A}=K-1\\ df_{E}=N-K \end{array} \end{array}$$

where *K* and *N* are the number of groups (*K* = 2) and total number of samples (*N* = 404), respectively.

When *F* < 1, the smaller the F value is, the smaller the difference of the feature between the two groups is, the worse the ability of the feature to recognize two kinds of proteins is; when *F* > 1, the larger the *F* value is, the greater the difference of the feature between the two groups is, the better the ability of the feature to recognize proteins is. Each F value corresponds to a *P*-value. The larger the *F*-value is, the smaller the *P*-value, that is, the greater the difference of the feature between groups.

The larger the F value is, the better the discriminant ability of the feature is. Therefore, all features can be sorted according to their F values, and the number of optimal feature subsets can be determined by incremental feature selection. The first feature subset is the feature with the highest median value in ranking. When the second highest value is added, a new feature subset is generated. This process was repeated from the higher F to the lower F value until all candidate features were added, therefore, for each sample, 4,000 feature subsets will be generated. The ε-th feature subset is composed of ε ranked *g*_*h*_-gap dipeptides and can be expressed as (Lin et al., 2015):

$$\begin{array}{l} P_{\varepsilon }={\left[f_{1}^{g_{h}},f_{2}^{g_{h}},\cdots \;,f_{\varepsilon }^{g_{h}}\right]}^{T},\;1\leq \varepsilon \leq 4,000,\;1\leq g_{h}\leq \;9 \end{array}$$

### Normalization

In machine learning, normalization of feature data is an important step. Because the characteristic information of protein sequence transformation is dimensionless, data normalization is used to facilitate the comparison and weighting of indicators of different scales. The data normalization can improve the convergence speed and the prediction accuracy of the model. The data normalization method used in this paper is MinMAXScaler, which normalizes each feature into [0,1] interval. The normalization function as follows:

$$\begin{array}{l} f_{u}^{{g_{h}}^{{\;}^{*}}}=\frac{f_{u}^{g_{h}}-{f_{u}^{g_{h}}}_{\min}}{{f_{u}^{g_{h}}}_{\max}-{f_{u}^{g_{h}}}_{\min}} \end{array}$$

### Support Vector Machine

In order to facilitate the comparison with the existing work, support vector machine (SVM) (Kumar and Panwar, 2011; Lin et al., 2015; Lai et al., 2017) is selected as the classifier in this work. The basic idea of SVM is to find an optimal classification hyperplane, which maximizes the interval between different types of samples. Kernel functions include linear and Gaussian kernels. In this paper, we use the radial basis function (RBF) (Cai et al., 2002; Yu et al., 2003; An et al., 2016). In this work, the parameters are tuned by the method of grid search-GridSearchCV (Liu et al., 2014). Grid search finds the optimal parameter combination by searching the specified parameter range exhaustively and gets the model performance results of each group of parameters combination. The search spaces for *C* is [10^−3^, 10^4^]. The search spaces for γ is [10^−4^, 10^5^]. Finally, the optimal combination of parameters [*C*, γ] is [1,1].

### Nested Cross-Validation Test

An important purpose of model validation is to select the most suitable model. A good model needs strong generalization ability to unknown data. This step of model validation can reflect the performance of different models for unknown data. In our method, we select the cross-validation model (Metfessel et al., 1993). Cross-checking divides the data set into two parts: training set and test set. Training set is used for model training, and test set is used to measure the prediction ability of the model. It can effectively prevent model over-fitting, and effectively evaluate the generalization ability of the model for data sets independent of training data.

Because the feature dimension in this paper is higher than 4,000, we chose nested cross-validation to prevent model overfitting. The samples are randomly divided into 10 equal and disjoint subsets in the external cycle of cross-validation. Nine of them are in turn selected as training sets, and one test subset is left, and then 10-fold cross-validation is carried out on the training set in the internal cycle. The internal loop performs feature selection and parameter optimization, and the external loop test set performs model performance evaluation. In nested cross-validation, the estimated true error is almost the same as the result obtained on the test set.

### Performance Assessment

The following indicators are used to evaluate the classification performance of the model.

1. Accuracy: Correctly identify the proportion of samples in the total sample.
  $$\begin{array}{l} Acc=\frac{TP+TN}{TP+TN+FP+FN} \end{array}$$
2. Sensitivity: The proportion of cancerlectins samples correctly identified as cancerlectins.
  $$\begin{array}{l} S_{n}=\frac{TP}{TP+FN} \end{array}$$
3. Specificity: The proportion of non-cancerlectins samples correctly identified as non-cancerlectins.
  $$\begin{array}{l} S_{p}=\frac{TN}{TN+FP} \end{array}$$
4. ROC curve

ROC curve is called “receiver operating characteristic curve”. The ROC curve takes FPR as the horizontal axis and TPR as the vertical axis.

The area under the ROC curve is AUC. AUC value is between 0 and 1, and the closer the AUC value is to 1, the better the performance of the classifier is.

$$\begin{array}{l} FPR=\frac{FP}{FP+TN}\\ TPR=\frac{TP}{TP+FN} \end{array}$$

where TP (True positive) and TN (True negative) denote the number of correctly predicted cancerlectins and the number of correctly predicted non-cancerlectins, respectively; FN is the number of the cancerlectins incorrectly predicted as the non-cancerlectins and FP is the number of the non-cancerlectins incorrectly predicted as the cancerlectins, respectively.

## Results

### Prediction Performance

The protein sequence is represented by the fusion of g-gap dipeptide features. After feature transformation, all protein sequences are converted into a 404^*^4,000 feature matrix. After variance analysis, *F*-values of features are sorted in descending order, and then feature selection and parameter optimization are carried out in a nested cross validation.

**Figure 2:**
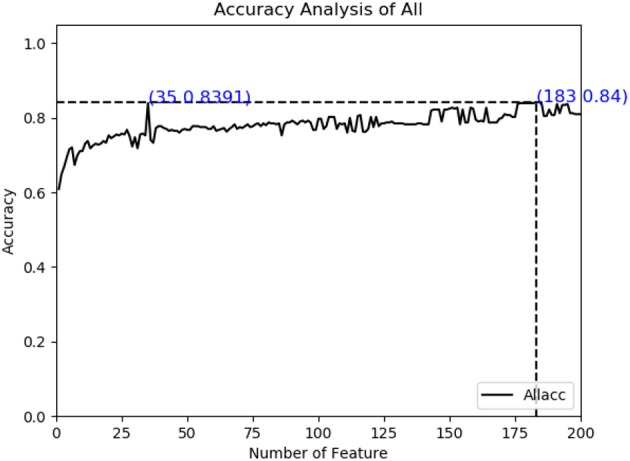
Prediction accuracy curve of feature subset.

As described in the feature extraction section, each sample sequence is transformed into a 4,000-dimensional dipeptide vector. Using too many low variance features to train prediction models will be relatively time-consuming, and it is possible to build over-fitting models. On the contrary, if the number of characteristic peptides is too small, they can only describe some properties of cancerlectins, even though each property may have a high variance and contain extremely rich information. Both of these conditions will lead to poor prediction results. The total number of protein sequence samples in data sets is 404. In order to build a reliable robust model, the number and accuracy of features need to be considered simultaneously. From Figure 2, it can be seen that the accuracy of feature subset increases slowly after 35 dimensions, until the number of feature subsets increases to 183 dimensions, the accuracy of model has small change from the feature subset of 35 dimensions. The accuracy of the first 183- dimensional model is 84% and that of the first 35-dimensional model of feature subset is 83.91%. Finally, the top 35 g-gap dipeptides are selected. Therefore, 35 g-gap dipeptides are selected as the optimal feature subset of the final classifier.

### Feature Description

As can be seen from Table 1, the variance of L_R1 is the largest, and the larger the variance, the smaller the *P*-value generally accompanied. The variance of L_R1 is 26.76446, *P*-value is 3.63E-07, Q_L1 variance is 11.06357, *P*-value is 0.000961. It can be seen that each feature in the optimal feature subset is significant and may play an important role in the classification and prediction of cancerlectins.

**Table 1:** *F*-value and *P*-value of features in optimal feature subset.

	***F*-value**	***P*-value**
L_R1	26.76446	3.63E-07
R_L4	24.81686	9.38E-07
Q_E0	20.28248	8.77E-06
I_D0	16.70216	5.28E-05
N_K3	16.34925	6.32E-05
N_D6	15.78628	8.40E-05
Q_P9	15.52386	9.61E-05
I_D4	15.23462	0.000111
D_N0	14.73123	0.000144
P_A1	14.28921	0.000181
N_D1	14.13802	0.000195
P_L5	13.87658	0.000223
L_P7	13.82865	0.000229
S_N5	13.69697	0.000245
A_L2	13.26494	0.000306
A_R2	12.96445	0.000357
L_P5	12.90963	0.000367
R_Q3	12.90722	0.000368
L_R8	12.702	0.000409
N_D3	12.40946	0.000476
N_G8	12.37352	0.000485
D_N7	12.2193	0.000526
D_N8	12.09143	0.000562
L_C0	11.94945	0.000605
N_V1	11.87518	0.000629
E_L5	11.79776	0.000655
Q_P1	11.78632	0.000659
Q_A0	11.54244	0.000748
L_E6	11.50195	0.000764
R_P4	11.4276	0.000794
P_L6	11.23968	0.000877
Q_M7	11.22643	0.000883
D_G0	11.22351	0.000884
S_P2	11.17902	0.000905
Q_L1	11.06357	0.000961

**Figure 3:**
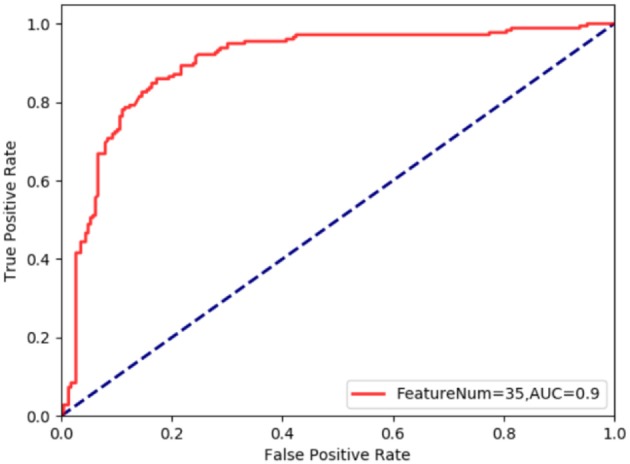
The ROC curve for cancerlectin prediction using the optimal 35 g-gap dipeptide.

As can be seen from Figure 3, the AUC of cancerlectin prediction using the optimal 35 g-gap dipeptide is 0.9, it means the classification performance of this classification model is good.

### Comparison With Existing Methods

In order to verify whether the classification model constructed in this work is over-fitting, 30 cancerlectins sequences were selected from NCBI database which were newly stored after 2012. From Table 2, prediction result 1 means correct classification, 0 means wrong classification. We can see there are 25 cancerlectins in new data were correctly predicted, the prediction accuracy of the new data is 83.3%.

**Table 2:** Classification of new data.

**ID**	**Prediction results**
1016841179	1
1016841154	1
1016841024	1
1016841005	1
560189093	1
720063203	1
727346123	1
469469047	0
403420575	1
385719187	1
384367986	1
388890228	1
1508736536	1
873090602	1
1022943309	1
974005177	1
392996940	0
385719190	1
1391723745	1
400260732	1
1370479176	1
1370451719	1
1034557774	1
768011769	1
768007991	1
768006291	0
1258501064	0
1272616377	1
1272616369	1
859066280	0

As can be seen from Table 3, the model in this work has better classification performance on new data, that is, the model generalization ability in this work is stronger.

**Table 3:** Comparison of classification results of new data.

**Methods**	**Acc (%)**
CancerPred (Amino acid composition) (Kumar and Panwar, 2011)	70
CancerPred (Dipeptide composition) (Kumar and Panwar, 2011)	76.67
CancerPred [Split composition (2-part)] (Kumar and Panwar, 2011)	56.67
CancerPred [Split composition (4-part)] (Kumar and Panwar, 2011)	60
Our Method	83.3

Comparing our method with other published methods, as shown in Table 4, the accuracy of the model obtained by our method is higher than that of previous studies. Though the specificity of our method is not much improved compared with Lin et al. (2015) and Lai et al. (2017), the sensitivity is greatly improved compared with the other three methods. The classification model improves the ability of correct recognition of cancer agglutinin samples, which shows that the classification model in this paper is effective.

**Table 4:** Comparison with the results of existing classification models.

**Method**	**S_n_ (%)**	**S_p_ (%)**	**Acc (%)**
Kumar and Panwar (2011)	68.00	69.90	69.09
Lin et al. (2015)	69.10	80.10	75.19
Damodaran et al. (2008)	75.28	80.53	77.48
Our method	83.15	80.87	83.91

## Discussion and Conclusions

Accumulated experimental evidences have shown that the classification of cancerlectins has important theoretical and practical significance for understanding its structural and functional characteristics, identifying drug targets, discovering tumor markers, and cancer treatment. More and more evidences show that it is crucial to propose an effective computational model to identify cancerlectins. In this paper, we developed a method based on the feature extraction algorithm of fusing g-gap dipeptide components to extract protein sequence features. Our method improve the feature extraction algorithm of protein sequence in cancerlectins prediction. We use the feature extraction algorithm of fusing g-gap dipeptide components to extract protein sequence features, which obtain an optimal feature subset containing 35 features. The accuracy, sensitivity and specificity are 83.91, 83.15, and 80.87% respectively. The results are better than those of the published methods. We also collect 30 new data form NCBI for predicted the performance of our method, and the prediction accuracy is 83.3%. Experimental results demonstrate that the performance of our method is better than the state-of-the-art methods for predicting cancerlectins.

Although our method can improve the prediction accuracy, it still has some limitations. Firstly, the benchmark dataset we used is relatively small, so there are some gaps in the data, and some specific attributes may be missing. Secondly, the extraction of protein sequence feature information is a key step in protein prediction. How to construct a better feature extraction algorithm remains to be further studied. Third, we only focus on the prediction of cancerlectin classification, how to choose a better classifier is our future work.

## Data Availability Statement

Publicly available datasets were analyzed in this study. This data can be found here: http://proline.physics.iisc.ernet.in/cgi-bin/cancerdb/input.cgi; http://www.uniprot.org/.

## Author Contributions

LQ and GH contributed to the conception and design of the study and developed the method. LQ and YW implemented the algorithms and analyzed the data and results. GH gave the ideas and supervised the project. LQ wrote the manuscript. GH and YW reviewed the final manuscript. All authors read and approved the final manuscript.

### Conflict of Interest

The authors declare that the research was conducted in the absence of any commercial or financial relationships that could be construed as a potential conflict of interest.
